# Population Status and Recovery Potential of *Dalbergia odorifera* Under Anthropogenic Disturbance: Evidence From Hainan Island, China

**DOI:** 10.1002/ece3.73843

**Published:** 2026-06-19

**Authors:** Chumin Ye, Kai Zhang, Yukai Chen, Yongkang Dai, Liu Chen

**Affiliations:** ^1^ Ministry of Education Key Laboratory for Ecology of Tropical Islands, Key Laboratory of Tropical Animal and Plant Ecology of Hainan Province, College of Life Sciences Hainan Normal University Haikou People's Republic of China

**Keywords:** anthropogenic disturbance, conservation strate, *Dalbergia odorifera*, population dynamics, population structure, survival analysis

## Abstract

To investigate the survival status and population dynamics of wild populations of *Dalbergia odorifera* on Hainan Island, we adopted the non‐induced interview method combined with stratified typical sampling surveys of 15 typical 20 m × 20 m quadrats, using standardized diameter at breast height (DBH)‐based age class division as the foundational grouping basis, supplemented by multiple analytical population demographic methods such as static life table analysis. Results show that although the population is of growing type (young individuals account for 77.36%), there are structural gaps and false growth caused by the lack of elderly individuals. The survival curve approximates the Deevey Type II, with the second and fourth age classes as mortality peaks. The population is disturbance‐sensitive but has certain recovery potential. The study identified dual bottlenecks: high mortality of young individuals and loss of middle‐aged and elderly individuals due to illegal logging. Accordingly, a phased protection strategy is proposed—improving habitats for young individuals, strengthening patrols to prevent illegal logging for middle‐aged and elderly individuals, and conducting long‐term dynamic monitoring—providing theoretical support for the protection and sustainable utilization of this species' resources.

## Graphical abstract

To explore the survival status and dynamics of wild populations of *Dalbergia odorifera* on Hainan Island, this study adopts field sampling combined with demographic analysis such as age class division and life table analysis. Results show that although the population is of growing type with abundant young individuals, there are structural gaps and false growth caused by the lack of mature trees. The survivorship curve approximates the Deevey‐II type, with two distinct mortality peaks at the second and fourth age classes. The population is disturbance‐sensitive but has certain natural recovery potential. The study identified dual bottlenecks: high mortality of young individuals and loss of middle‐aged and mature trees due to illegal logging. Accordingly, targeted stage‐specific conservation strategies are proposed—improving habitats for young individuals, strengthening patrols to prevent illegal logging for middle‐aged and elderly individuals, and conducting long‐term dynamic monitoring—providing scientific support for the protection and sustainable utilization of this species' resources.

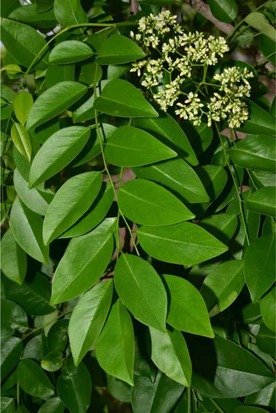

## 1 Introduction

A population refers to a collection of conspecific individuals within a specific time and space. It is the basic unit for the survival, development, and evolution of species, and also a key link connecting individual organisms, communities, and ecosystems (Zhao et al. 2020). In plant communities, population size largely affects the temporal dynamics of their maintenance and development (Tian et al. 2020). The core research of population ecology focuses on population structure and quantitative dynamics (Zhang et al. 2014), and population demography provides key scientific methodologies for this purpose, including age structure analysis, static life table construction, survivorship curve plotting, survival function analysis, and time series prediction (Li et al. 2022; Zhang et al. 2022). Analyzing plant population structure and dynamic changes through these methods can not only objectively reflect the historical dynamic characteristics and maintenance mechanisms of plant populations but also predict their future development trends. This helps identify factors limiting plant population regeneration, explore the endangered mechanisms of species from a population ecology perspective (Li et al. 2022; Xiao et al. 2025), and is of great significance for the protection, regeneration, and ecological restoration of plant resources (Ge et al. 2025).

**FIGURE 1:**
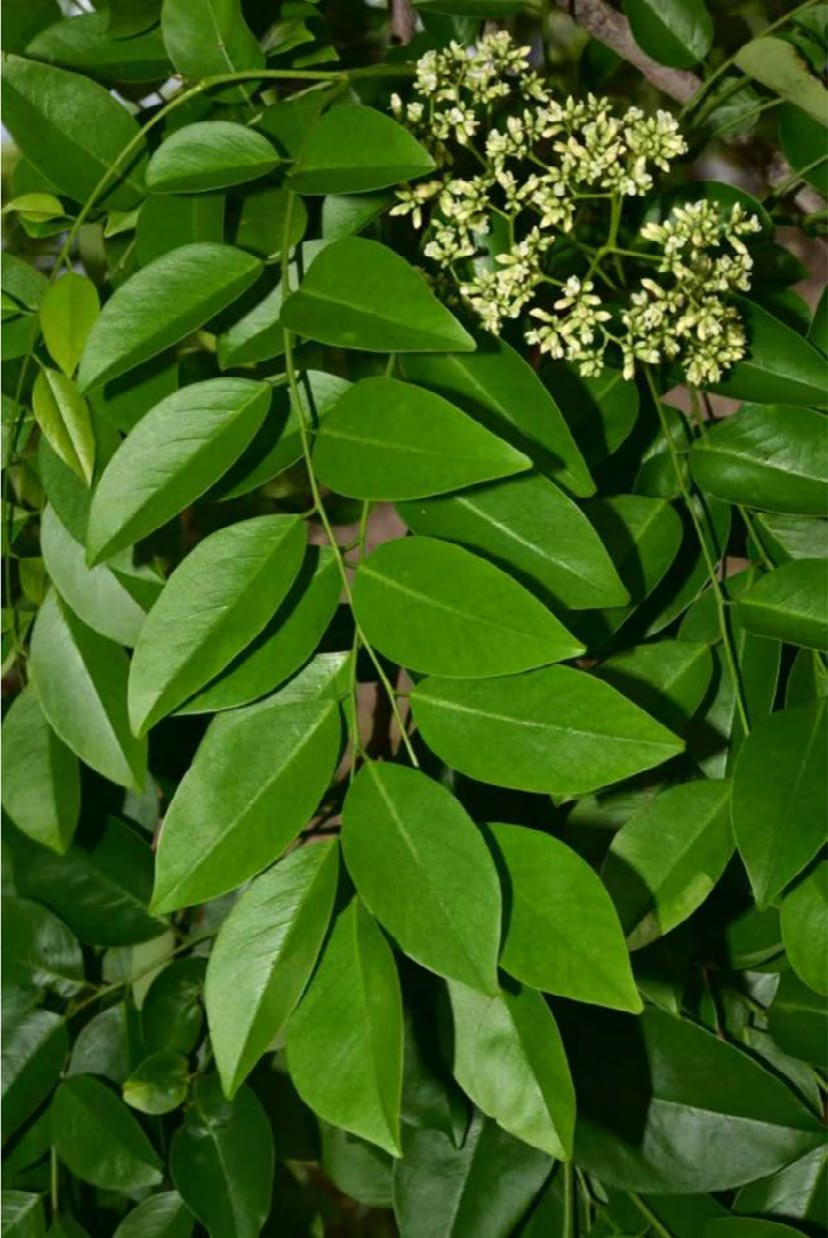
*D. odorifera* flower and leaf photos.

*Dalbergia odorifera* T. C. Chen, an endemic arbor of the genus *Dalbergia* in Fabaceae, is a national second‐class key protected wild plant in China, with important ecological, medicinal, and other economic values (Figure 1). Its wood can be used for furniture making, and the heartwood “Jiangxiang” (fragrant rosewood) from its roots is a traditional medicinal material for analgesia and hemostasis. It is also a key strategic resource and precious spice tree species in China (Zhang et al. 2024). However, due to the extremely high commercial value of its heartwood, it has suffered unreasonable exploitation by humans, leading to a sharp reduction in its natural resources (Liu et al. 2008), and even making it difficult to trace. In addition, the formation of heartwood in this species is slow under natural conditions (starting to form at about 7 years old and maturing at more than 30 years old), which is the core bottleneck restricting its utilization as a resource (Huang 2022; Lin et al. 2024). Current studies have covered areas such as stress response (Qiu et al. 2016; Zhou et al. 2025), DNA molecular identification (Tang et al. 2013; Yu 2015), chemical composition analysis (Ma 2018; Li, Li, and Li 2018), and cultivation and propagation (Li, Liu, et al. 2018; Luo et al. 2022; Chen et al. 2024) of *D. odorifera*. Most of these studies focus on cultivated individuals and have not formed a systematic connection with the contradiction between the protection and sustainable utilization of wild resources of this species. In particular, the natural resources and survival status of wild *D. odorifera* urgently need in‐depth investigation. Based on this, this study takes *D. odorifera* as the research object and explores the following through analyzing the age structure, survivorship curve, and static life table of wild *D. odorifera* populations: (1) the age structure and dynamic characteristics of existing wild *D. odorifera* populations; (2) the future recovery potential and limiting factors of *D. odorifera* populations. The aim is to analyze the endangered status and population maintenance mechanisms of *D. odorifera* from a population ecology perspective, and provide a scientific basis for formulating protection strategies, management, and rejuvenation of the natural resources of this species.

## 2 Materials and Methods

### 2.1 Overview of the Study Area

Hainan Island is located at the southernmost tip of China, as the second‐largest island in the country, with geographical coordinates between 18°10′~20°10′ N latitude and 108°37′~111°03′ E longitude. The climate of Hainan Island is a typical tropical marine monsoon climate, characterized by warmth and humidity throughout the year. The annual average temperature is maintained at 22°C~26°C, with the average temperature in January being 16°C~21°C and in July being 25°C~29°C. The annual sunshine duration ranges from 1750 to 2650 h, and the annual precipitation is 1500–2500 mm. Precipitation is unevenly distributed seasonally: the rainy season from May to October accounts for 70%~90% of the annual total, while November to April of the following year is the dry season. The vertical distribution of soil is obvious: laterite soil is widely developed in areas below 300 m above sea level, with significant aluminization characteristics, being acidic and having high nutrient content; mountain red soil is dominant in mountainous areas between 300~800 m. Vegetation types show typical vertical zonation: tropical monsoon forests are distributed in hilly platforms below 500 m, tropical rainforests develop in mountains between 500~1000 m, and mountain rainforests, evergreen broad‐leaved forests, and mountain top dwarf forests appear sequentially with increasing altitude.

### 2.2 Survey Methods

In the early stage, we systematically clarified the complete natural distribution range of *D. odorifera* on Hainan Island by combing through herbarium specimens, historical literature, and Hainan Provincial Forestry Survey Bulletins, combined with non‐induced interviews with villagers, herbal collectors, forest rangers, and other groups with mountain entry experience to record all historical and existing potential distribution points, which were then verified one by one through field surveys led by the interviewees. All sampling was strictly conducted within this confirmed natural distribution range, and a stratified typical sampling scheme was adopted with the core objective of fully covering the distribution area and objectively reflecting the true survival status of the population.

**FIGURE 2:**
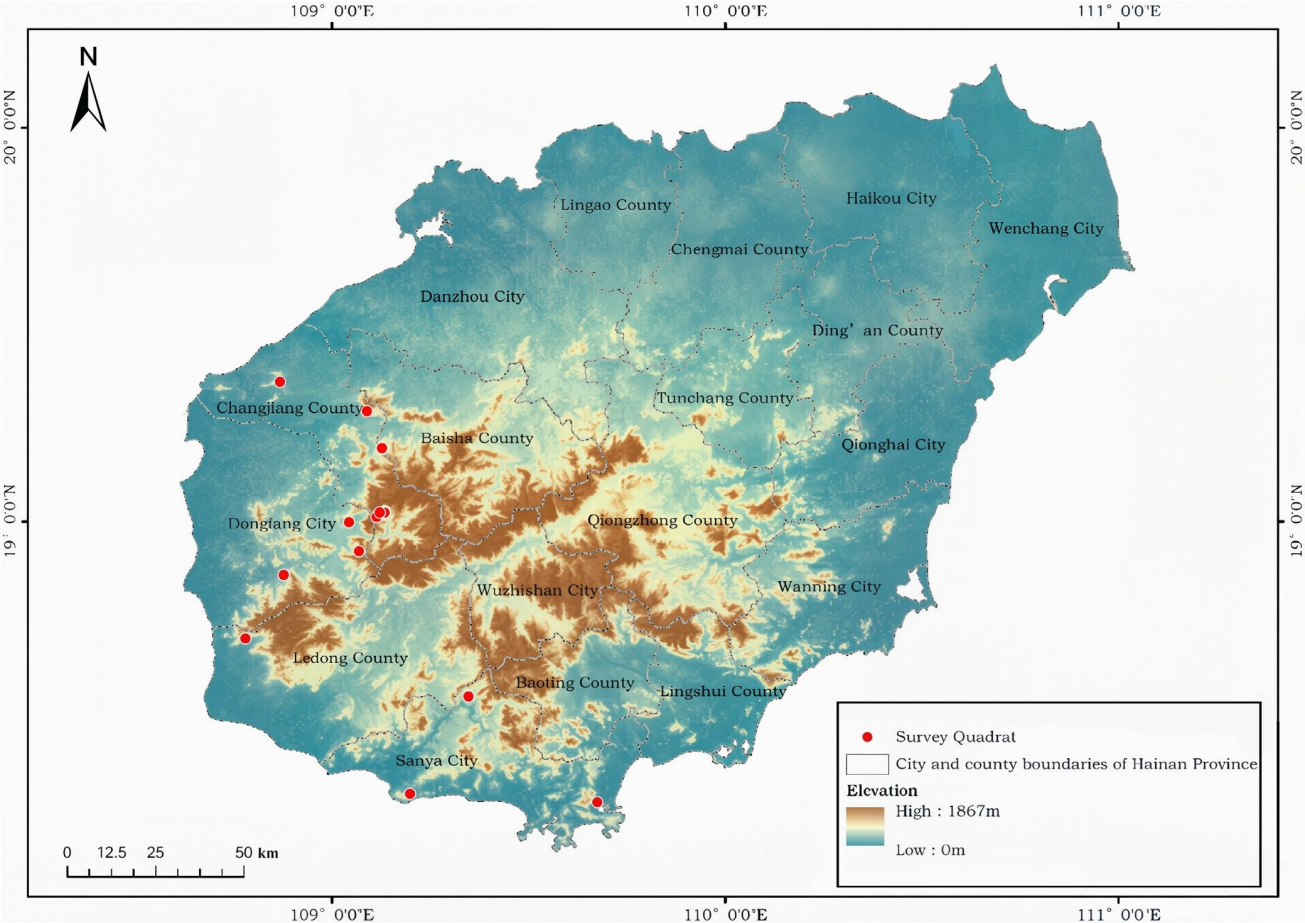
*D. odorifera* survey quadrat distribution map.

A total of 15 typical 20 m × 20 m quadrats were set up, including 8 core demographic quadrats with intact existing populations and 7 disturbed control quadrats with only illegal logging and root‐digging traces (for analyzing anthropogenic disturbance regimes and their impacts on population survival and structure) (Figure 2). The sampling design was formulated based on four core dimensions: (1) Coverage representativeness: The 15 quadrats cover more than 90% of the existing natural distribution points, all geographical units and the entire natural elevation range (50–1000 m) of *D. odorifera* on Hainan Island, with samples set at all investigable concentrated distribution points, ensuring sufficient sample representativeness; (2) Sampling method compliance: The stratified typical sampling method conforms to the *Technical Specification for Investigation of Wild Plants with Extremely Small Populations* (LY/T 2934.1–2018), adapts to the endangered and scattered distribution characteristics of the species, avoids empty sample bias caused by whole‐region random sampling, and refers to the general paradigm for rare tree surveys in the same region of Hainan; (3) Quadrat specification and quantity rationality: The 20 m × 20 m quadrat size complies with the National Standard for Forest Inventory (GB/T 33027–2016), and the number of quadrats is consistent with peer‐reviewed studies on endangered tree species in the same region, which can meet the statistical requirements for population structure, life table and other demographic analyses; (4) Gradient setting comprehensiveness: A full elevation gradient and a two‐level anthropogenic disturbance gradient were set to completely cover the natural habitats and disturbance scenarios of the species, enabling systematic analysis of the effects of habitat characteristics and disturbance on population dynamics.

During the survey, detailed records were made of the number, DBH (or ground diameter for seedlings < 1.3 m in height), plant height and crown width of *D. odorifera* within the quadrats, as well as habitat factors such as altitude, slope, aspect and canopy density of the sample plots (Table 1). For individuals outside the quadrats, relevant indicators were recorded by actual measurement. All data provided a basis for subsequent population structure analysis, exploration of endangered causes and formulation of protection strategies.

**TABLE 1:** Basic information of core demographic quadrats and disturbed control quadrats of *D. odorifera* survey plots.

Plot no.	Location	Elevation (m)	Aspect	Slope (°)	Canopy density (%)
1	Dongfang Daguangba	183	North	21	68
2	Dongfang Daguangba	94	Northwest	5	70
3	Changjiang Exianling	519	Northwest	26	65
4	Changjiang Exianling	715	North	5	26
5	Changjiang Exianling	1001	North	36	76
6	Changjiang Exianling	454	North	22	80
7	Changjiang Baomeiling	513	South	5	73
8	Changjiang Baomeiling	507	South	5	73
9	Changjiang Mihouling	330	Northeast	18	80
10	Changjiang Shiyuetian	112.8	Flat	5	80
11	Sanya Nanshanling	140.3	Northeast	16	65
12	Sanya Yucai	314.8	Northwest	33	70
13	Ledong Jianfengling	234	East	21	80
14	Baisha Ruwengling	268.5	East	12	90
15	Sanya Zhuluoling	51	South	22	65

### 2.3 Data Analysis Methods

Based on field survey data, this study systematically investigated the wild population of *D. odorifera* using multidisciplinary analytical approaches integrated with professional software. Details are as follows:

Age Class Division Due to the impracticality of direct age determination for wild *D. odorifera* populations, the widely adopted “space‐for‐time substitution method” (Gui, Ye, et al. 2025) was employed. Previous studies have confirmed that DBH and heartwood diameter of *D. odorifera* increase positively with tree age, with a highly significant linear correlation between DBH and tree age and an average annual DBH increment of approximately 0.8 cm under natural habitats (Chen et al. 2016; Zhou et al. 2022). Populations were categorized into 5 age classes based on 2 cm intervals of diameter at breast height (DBH): Class I: (0, 2) cm, Class II: (2, 4) cm, Class III: (4, 6) cm, Class IV: (6, 8) cm, and Class V: > 8 cm. It should be acknowledged that age class division based on DBH may introduce three types of potential errors: (1) habitat heterogeneity; (2) biotic and abiotic stresses (e.g., liana coverage, interspecific competition, drought); and (3) anthropogenic disturbances (e.g., illegal logging, pruning), which may cause mismatches between DBH and actual age. To objectively reflect the true survival state of the population, this study included 100% of all growth‐suppressed individuals (including those with liana entanglement, understory low light stress, interspecific competition, resprouting after illegal logging, and pest/disease damage) without any exclusion his age class system provided the unified grouping framework for all subsequent population demographic analyses, including static life table construction, survivorship curve plotting and survival function calculation. An age structure diagram was constructed to visualize the population age distribution.

Static Life Table and Survival Curve. A static life table was constructed following the methodology of Jiang (1992), using the actual number of individuals in each age class without applying smoothing corrections to preserve the authenticity of ecological processes. Survival curves were plotted with age classes as the x‐axis and the logarithm of standardized survival numbers as the y‐axis. Curve types were classified according to Deevey's three classic survival curve models and validated using the exponential function (y = a_0_e^−bx^) and power function (y = a_0_x^−b^) proposed by Hett (Hett and Loucks 1976). Additionally, curves of mortality rate (*q*_x_) and disappearance rate (*K*_x_) were generated for supplementary analysis.

Sensitivity analysis. To test whether the mortality and hazard rate estimates in the static life table might be biased due to the small sample size in older age classes, a sensitivity analysis was performed using standardized regression coefficients. A linear regression model was constructed with the natural logarithm of standardized survivors (*LNl*_x_) as the dependent variable and age class (*x*) as the independent variable. The standardized regression coefficient (Beta) and its significance were calculated, and multicollinearity was tested using the variance inflation factor (VIF). This analysis was used to evaluate the strength of the effect of age class on population survival dynamics and the robustness of the conclusions.

Following Chen's (1998) quantitative framework for plant population dynamics, we calculated three key indices: (1) inter‐age‐class dynamic index (*V*_*n*_) to reflect individual number changes between adjacent age classes; (2) overall population dynamic indices under undisturbed (*V*_*pi*_) and disturbed (*V*_*pi*_ *'*) conditions; and (3) maximum disturbance risk probability (*P*_max_). The precise calculation formula of *P*_max_ was derived from Chen (1998):

$$P_{\max}=1/k\cdot \min\left(S_{1},S_{2},S_{3},\ldots ,S_{k}\right)$$

where *k* is the total number of age classes divided for the population (*k* = 5 in this study, corresponding to the 5 DBH‐based age classes). This index represents the maximum risk tolerance threshold of the population to random external disturbances: a larger value indicates higher sensitivity to anthropogenic disturbances and weaker anti‐interference ability. These indices collectively characterize the population's structural dynamics and sensitivity to external disturbances.

Survival Analysis Survival patterns of the population were analyzed using four core functions from Yang Fengxiang (Yang et al. 1991) survival analysis theory: survival function (*S*_x_), cumulative mortality function (*F*_x_), hazard rate function (*λ*_x_), and death density function (*f*_x_). Corresponding curves were plotted with age classes as the x‐axis and function values as the y‐axis to illustrate temporal variations in survival and mortality risks.

Time Series Prediction. The simple moving average method, a robust tool for population trend forecasting (Li et al. 2022), was used to simulate and predict the number of individuals in each age class for the next 2, 3, 4, and 5 age‐class intervals. Population dynamics analysis using matrix model. The population dynamics of *D. odorifer*a were analyzed using the Lefkovitch stage‐structured projection matrix model (Caswell 2001). Based on the number of surviving individuals in five age classes from field surveys, a survival‐growth transition probability matrix was constructed, combined with fecundity data for each age class (fecundities for age classes III, IV, and V were 0.240, 0.320, and 0.430 respectively, while those for classes I and II were 0). The finite population growth rate *λ* was obtained as the dominant eigenvalue of the matrix to assess population trends. Elasticity analysis was used to quantify the relative contributions of different life‐history processes to population growth.

Data Processing: All statistical analyses (including the moving average and matrix model analyses) were performed using Microsoft Excel 2019 and SPSS Statistics 26, and R 4.5.1. Graphical visualization (including age structure diagrams, survival curves, and function curves) was conducted with Origin 24.

## 3 Results

### 3.1 Population Structure Analysis

**FIGURE 3:**
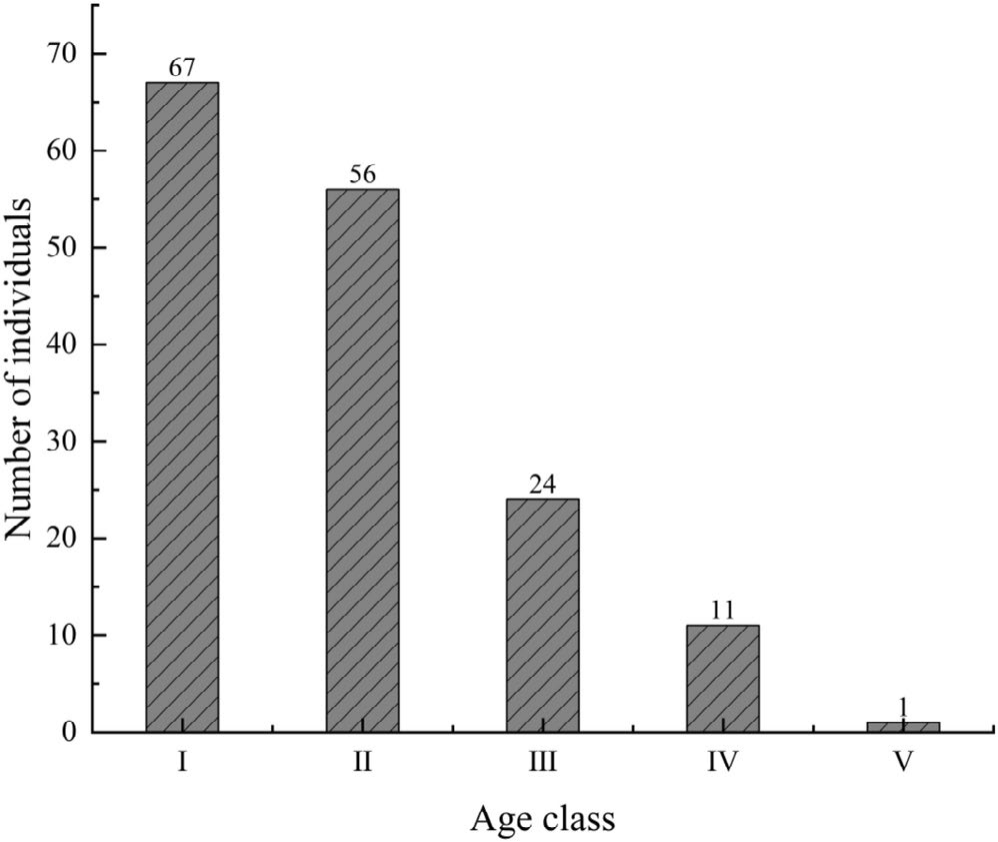
Age structure of *D. odorifera* population.

A total of 159 individuals of *D. odorifera* were recorded in this survey, with the largest DBH of 12 cm. The survey found that *D. odorifera* is mainly distributed in tropical monsoon forests in Hainan. The population age structure diagram was drawn with age classes (I, II, III, IV, V) as the abscissa and the number of individuals as the ordinate. As shown in Figure 3, the population structure of *D. odorifera* shows a trend of gradual decrease in the number of individuals with increasing age classes. Among them, Age class I has the largest number of individuals (67), followed by Age class II (56), Age class III (24), Age class IV (11), and Age class V has the smallest number of individuals (only 1). From the perspective of age structure type, young age classes (I, II) account for a large proportion, about 77.36% of the total, while old age classes (IV, V) account for a small proportion, about 7.56% of the total. The overall age structure of the population tends to be an increasing population.

### 3.2 Static Life Table and Survivorship Curve of the Population

#### 3.2.1 Static Life Table

As shown in Table 2, with the increase of age classes, the standardized survival number (*lx*) and the logarithm of standardized survival number (*LNlx*) decrease step by step; the number of dead individuals (*dx*) is the largest in Age class II (478), while the number of deaths in other age classes is basically maintained at a consistent level; the mortality rate (*q*_*x*_) shows an increasing trend with the increase of age classes, forming two death peaks in Age classes II and IV, with the highest mortality rate (0.91) in Age class IV; the life expectancy (*ex*) reflects the survival ability of individuals in the age class. It can be seen from the life table that the life expectancy decreases with the increase of age classes and is the largest (1.87) in Age class I; the attrition rate (*K*_*x*_) of the population shows an increasing trend, reaching 2.40 in Age class IV, while the survival rate (*S*_*x*_) shows a decreasing trend with the increase of age classes. This static life table indicates that young individuals in Age class II and old individuals face great survival pressure, and the number of individuals in old age classes (V) is extremely small. One possible reason for the scarcity of old individuals in *D. odorifera* is their high vulnerability to illegal logging and theft, as the highly valuable heartwood forms at older ages, making these trees a target for poachers.

**TABLE 2:** Static life table of *D. odorifera* population.

Age class	*a*_*x*_	*l*_*x*_	*LNl*_*x*_	*dx*	*q*_*x*_	*Lx*	*T*_*x*_	*e*_*x*_	*K*_*x*_	*S*_*x*_
I	67	1000	6.91	164	0.16	918	1873	1.87	0.18	0.84
II	56	836	6.73	478	0.57	597	955	1.14	0.85	0.43
III	24	358	5.88	194	0.54	261	358	1.00	0.78	0.46
IV	11	164	5.10	149	0.91	90	97	0.59	2.40	0.09
V	1	15	2.70	0	0.00	7	7	0.50	0.00	0.00

*Note: a*_*x*_ = number of surviving individuals in age class *x*; *l*_*x*_ = standardized number of surviving individuals in age class *x*; *d*_*x*_ = standardized number of dead individuals between age class *x* and *x* + 1; *q*_*x*_ = mortality rate between age class *x* and *x* + 1; *L*_*x*_ = average number of surviving individuals between age class *x* and *x* + 1; *T*_*x*_ = total number of individuals from age class *x* to beyond age class *x*; *e*_*x*_ = life expectancy of individuals entering age class *x*; *LNl*_*x*_ = logarithm of standardized number of surviving individuals; *K*_*x*_ = attrition rate of age class *x*; *S*_*x*_ = survival rate of age class *x*.

To test whether the static life table might lead to overestimation of late‐stage mortality and hazard rates due to small sample size (especially only one individual in age class V), we performed a standardized regression coefficient sensitivity analysis. The results showed that age class was a highly sensitive factor affecting population survival, with a standardized regression coefficient Beta = −0.932 (*t* = −4.438, *p* = 0.021 < 0.05). For each one‐level increase in age class, the logarithm of population survival decreased by an average of 1.005 units, indicating a significant negative correlation. The regression model had no multicollinearity issue (VIF = 1.000), confirming the stability and reliability of the results. This analysis confirms that, although the extreme mortality rates in age classes IV and V of the static life table may be influenced by small sample size, the conclusion that age class is the core factor regulating the survival dynamics of the wild *D. odorifera* population remains robust (Table 3).

**TABLE 3:** Sensitivity analysis results.

	Beta	*t*	P	Lower bound of the 95.0% confidence interval	Upper bound of the 95.0% confidence interval	VIF
Age class	−0.932	−4.438	0.021	−1.726	−0.284	1.000

#### 3.2.2 Survivorship Curve

**FIGURE 4:**
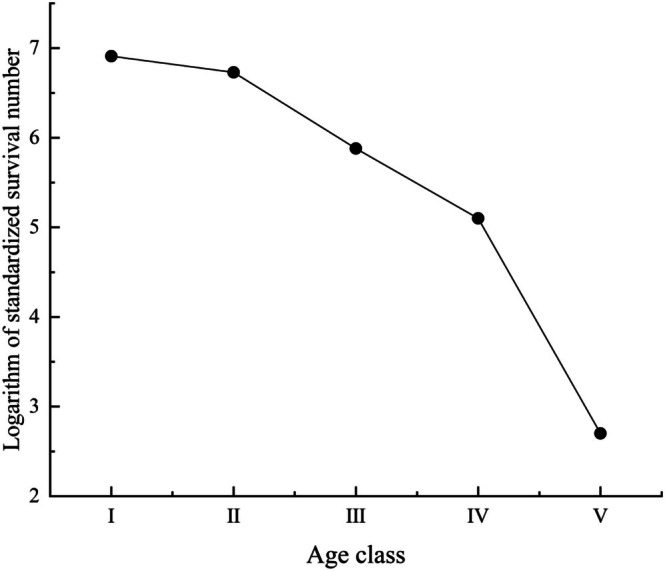
Survivorship curve of *D. odorifera* population.

The survivorship curve is based on the number of surviving individuals and can reflect the survival status of tree individuals in each age class (Li et al. 2014). In this study, the survivorship curve was drawn with the logarithm of standardized survival number (*LNl*_*x*_) of *D. odorifera* population as the ordinate and population age classes as the abscissa (Figure 4). As shown in Figure 2, the logarithm of standardized survival number of the population decreases relatively gently from Age class I to II, starts to show a steeper downward trend from Age class II to IV, and presents a sharp downward trend from Age class IV to V.

The exponential function y = a_0_e^−bx^ and power function y = a_0_x^−b^ were used to test the survivorship curve, and function fitting was performed using SPSS 26. As shown in Table 4, the *R* ^2^ and *F* values of the exponential function (0.785 and 10.97, respectively) are both greater than those of the power function (0.601 and 4.528, respectively), and the *p* value of the exponential function reaches a significant level (*p* = 0.045 < 0.05). Moreover, the SSE of the exponential function (0.127) is smaller than that of the power function (0.236). This indicates that the fitting result of the exponential function is better, and the population survivorship curve of *D. odorifera* is more consistent with the Deevey Type II. The model test results are basically consistent with the change trend of the survivorship curve.

**TABLE 4:** Test models of survivorship curves of *D. odorifera* population.

Survivorship curve	Fitting model	Equation	*R* ^2^	*F*	*p*	SSE
Deevey Type II	y = a_0_e^−bx^	y = 9.912e^−0.216×^	0.785	*F*(1, 3) = 10.97	0.045	0.127
Deevey Type III	y = a_0_x^−b^	y = 8.137×^−0.469^	0.601	*F*(1, 3) = 4.528	0.123	0.236

*Note:* Fitting was based on 5 age classes with sample sizes: I (67), II (56), III (24), IV (11), V (1). All age classes of the wild population were covered for reproducible results.

#### 3.2.3 Mortality Rate and Attrition Rate

**FIGURE 5:**
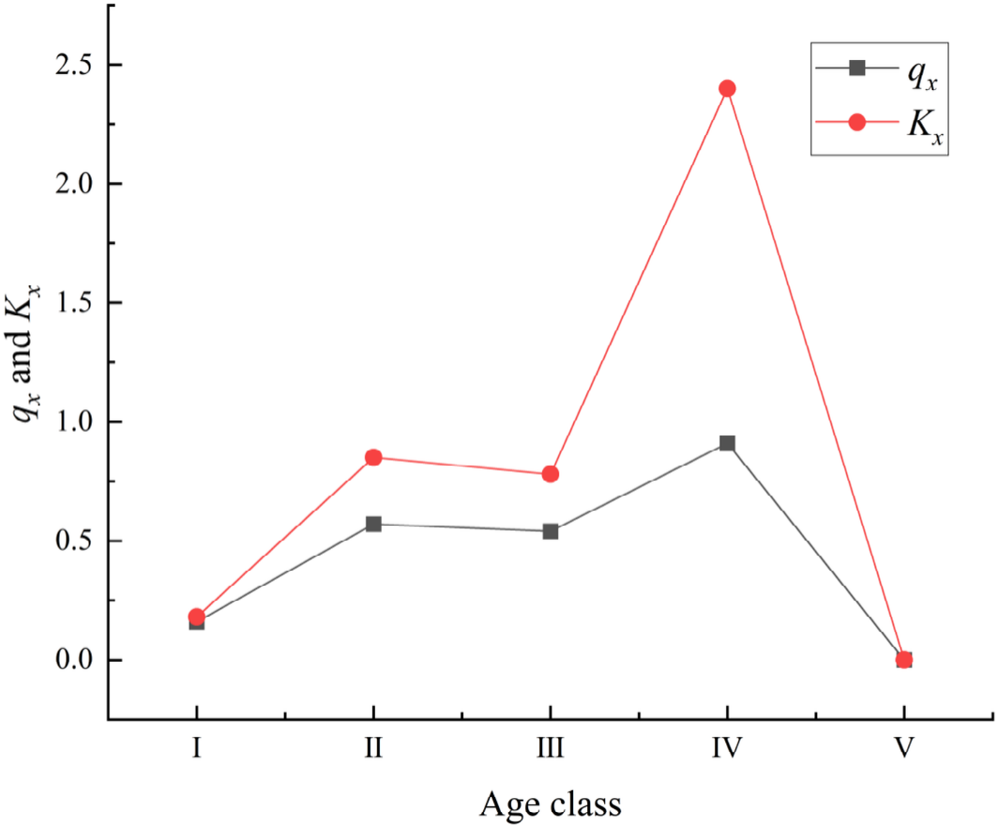
Curves of mortality rate (*q*_*x*_) and attrition rate (*K*_*x*_) of *D. odorifera* population.

As shown in Figure 5, the mortality rate (*q*_*x*_) shows a trend of first increasing and then decreasing with the change of age classes. It is low in Age class I, gradually increases from Age class II to IV, reaches the peak in Age class IV, and shows a significant downward trend from Age class IV to V; the fluctuation of the attrition rate (*K*_*x*_) is more intense. It is low in Age class I, increases significantly in Age class II, decreases slightly in Age class III, rises sharply to the peak in Age class IV, and drops rapidly to 0 in Age class V. Overall, the change range of the attrition rate is much larger than that of the mortality rate, and the attrition rate in Age class IV is much higher than the mortality rate in the same period, indicating that the intensity of individual disappearance (such as death or emigration) in this age class is higher. This is consistent with the analysis result of the survivorship curve.

### 3.3 Quantitative Analysis of Population Dynamics

The results of the quantitative analysis of *D. odorifera* population dynamics show (Table 5) that the quantity change indices (*V*_*1*_(16.42), *V*_*2*_(57.14), *V*_*3*_(54.17), *V*_*4*_(90.91)) between adjacent age classes are all positive, indicating that the number of individuals between adjacent age classes shows an increasing trend; the population structure dynamic index *V*_*pi*_ (41.77) and the corrected *V*_*pi*_' (8.35) are also positive, reflecting that the population shows an increasing structure when not affected by external interference. However, *V*_*pi*_ *'* is much smaller than *V*_*pi*_, indicating that the increasing trend of the population is not obvious when affected by external interference; in addition, the maximum risk probability *P*_*max*_ that the population bears against external interference is 20.00, indicating that *D. odorifera* population has a certain upper limit of risk bearing when facing interference, and is an interference‐sensitive population.

**TABLE 5:** Quantitative analysis results of age‐class dynamics of *D. odorifera* population.

Quantitative index (%)	*V*_*1*_	*V*_*2*_	*V*_*3*_	*V*_*4*_	*V*_*pi*_	*V*_*pi*_ *'*	*P*_*max*_
Analysis results	16.418	57.143	54.167	90.909	41.772	8.354	20.00

### 3.4 Survival Analysis

**FIGURE 6:**
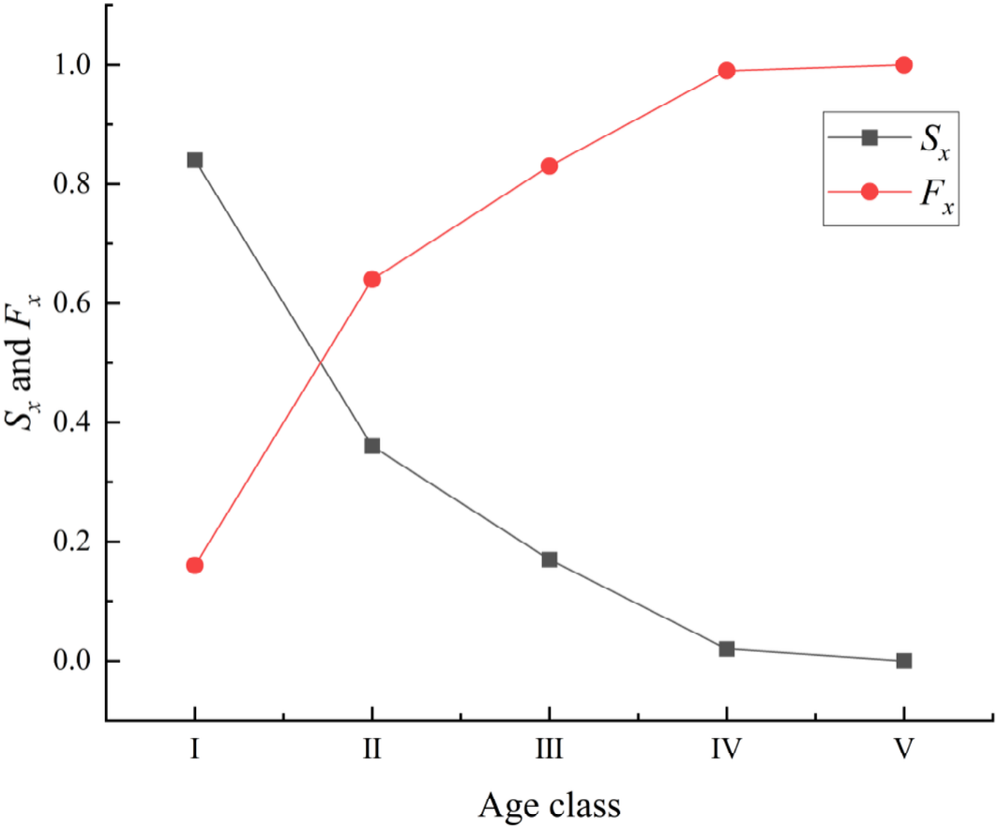
Curves of survival rate (*S*_*x*_) and cumulative mortality rate (*F*_*x*_) of *D. odorifera* population.

As shown in Figure 6, the survival function (*S*_*x*_) continuously decreases from Age class I to V. The survival rate is high in Age class I, decreases rapidly after Age class I~II, slows down from Age class II~IV, tends to be gentle from Age class IV~V, and is close to 0 in Age class V. This reflects that the probability of *D. odorifera* individuals surviving from low age classes to high age classes decreases sharply; the cumulative mortality function (*F*_*x*_) shows an increasing trend with the increase of age classes, presenting an inverse complementary relationship with the survival function. It continues to rise with the increase of age classes, with a low cumulative mortality rate (about 0.15) in Age class I, then gradually increases, and is close to 1.0 from Age class IV~V.

**FIGURE 7:**
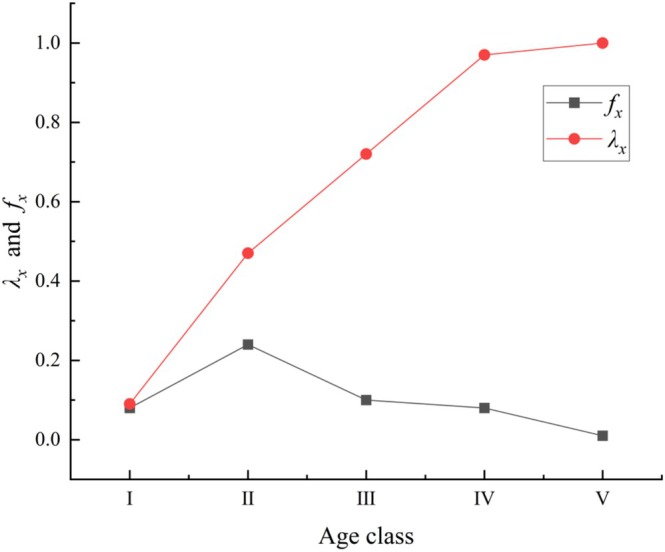
Mortality density (*f*_*x*_) and hazard rate (*λ*_*x*_) curve of *D. odorifera* population.

As shown in Figure 7, the mortality density (*f*_*x*_) shows a trend of first increasing and then decreasing. It is low in Age class I, rises to a small peak (about 0.25) in Age class II, then gradually decreases from Age class III, and is close to 0 in Age class V. This reflects the differences in the probability density of individual death per unit time in different age class intervals, among which the mortality density in Age class II is relatively the highest; the hazard rate (*λ*_*x*_) shows a continuous steep upward trend with the increase of age classes. The risk is low in Age class I, increases rapidly from Age class I~IV, is close to 1.0 in Age class IV, and reaches about 1.0 in Age class V. This indicates that the death risk faced by *D. odorifera* population individuals surviving from low age classes to high age classes increases sharply with age, and the immediate death risk of individuals in high age classes is extremely high. The hazard rate measures the relative probability of individual death in a specific age class.

### 3.5 Time Series Prediction

In this study, the moving average method was used for single translation to predict the quantity change trend of *D. odorifera* population after 2, 3, 4, and 5 age class periods (Table 6). After 2 age class periods (*M*_*2*_), the predicted number of individuals in Age class II is 62, slightly higher than the initial number (56) of Age class II; after 2 age class periods (*M*_*2*_) and 3 age class periods (*M*_*3*_), the number of individuals in Age class III is 40 and 49, showing a gradual upward trend; after 2 age class periods (M_2_), 3 age class periods (*M*_*3*_), and 4 age class periods (*M*_*4*_), the number of individuals in Age class IV is 18, 30, and 40, respectively, with the predicted value increasing with the number of periods; after 2 age class periods (*M*_*2*_), 3 age class periods (*M*_*3*_), 4 age class periods (*M*_*4*_), and 5 age class periods (*M*_*5*_), the number of individuals in Age class V is 6, 12, 23, and 32, respectively, showing a continuous upward trend with the increase of periods. Overall, the number of individuals in each age class of *D. odorifera* population shows an obvious upward trend, which is consistent with the results of the quantitative analysis of population dynamics. This indicates that under ideal conditions in the future, *D. odorifera* has the potential to recover to a certain population size.

**TABLE 6:** Dynamic time series prediction of *D. odorifera* population.

Age class	Number of individuals	*M*_*2*_	*M*_*3*_	*M*_*4*_	*M*_*5*_
I	67	—	—	—	—
II	56	62	—	—	—
III	24	40	49	—	—
IV	11	18	30	40	—
V	1	6	12	23	32

*Note: M*_*2*_, *M*_*3*_, *M*_*4*_, and *M*_*5*_ in the table represent the population size after 2, 3, 4, and 5 age class periods, respectively, with age classes corresponding to I–V in the table.

Based on the Lefkovitch projection matrix, the finite rate of increase (dominant eigenvalue) λ of the D. odorifera population was calculated to be 1.168, indicating that the population exhibits a significant increasing trend under current life history parameters, with an annual growth rate of 16.8%. This confirms that the population has strong natural recovery potential under suitable environmental conditions (Table 7). Elasticity analysis revealed that survival and growth processes contributed 90.7% to population growth, while fecundity contributed only 9.3%. Among these, stasis in age class IV (elasticity = 0.261) was the most critical process driving population growth, followed by stasis in age class V (elasticity = 0.186). The elasticity results demonstrated that the long‐term survival of mature individuals is the core factor sustaining the growth of *D. odorifera* populations. The population growth trend predicted by the projection matrix model was fully consistent with that from the moving average time series analysis, both showing a clear upward trend in the number of individuals across all age classes over time, which jointly verified that the D. odorifera population possesses favorable recovery potential (Table 8).

**TABLE 7:** *D. odorifera* population Lefkovitch projection matrix.

Age class (*t*) age class (*t* + 1)	I	II	III	IV	V
I	0.000	0.000	0.240	0.320	0.430
II	0.836	0.571	0.000	0.000	0.000
III	0.000	0.429	0.542	0.000	0.000
IV	0.000	0.000	0.458	0.909	0.000
V	0.000	0.000	0.000	0.091	1.000

*Note:* The matrix element *A*_*i,j*_ represents the contribution of individuals in age class *j* at time *t* to the number of individuals in age class i at time *t + 1*. The first row corresponds to fecundity terms, and rows 2–5 correspond to survival and growth terms.

**TABLE 8:** *D. odorifera* population elasticity matrix.

Age class (*t*) age class (*t* + 1)	I	II	III	IV	V
I	0.000	0.000	0.018	0.043	0.031
II	0.093	0.089	0.000	0.000	0.000
III	0.000	0.093	0.080	0.000	0.000
IV	0.000	0.000	0.074	0.261	0.000
V	0.000	0.000	0.000	0.031	0.186

*Note:* Larger values indicate a stronger influence of the corresponding life history process on the population growth rate.

## 4 Discussion

Population structure reflects plant adaptability and development trends, and studying the age structure of rare and endangered plants can reveal their survival status and predict population dynamics (Wang et al. 2023; Gui, Fang, et al. 2025). The *D. odorifera* population exhibits a growing type of age structure, with young‐aged individuals (classes I, II) accounting for 77.36%, while old‐aged individuals (classes IV, V) account for only 7.56%. The survival curve shows a gentle decline from class I to II, an accelerated decline from class II to IV, and a sharp decline from class IV to V; mortality and disappearance rates first increase and then decrease, peaking at class IV before dropping sharply. The individual numbers decrease by 45.8% from class III to IV and by 90.9% from class IV to V, reflecting a severe lack of old‐aged individuals and an incomplete population structure. Similar structures are found in *Horsfieldia hainanensis* and *Paranephelium hainanense* (Jiang et al. 2017; Luo et al. 2018). However, the sharp decline in old‐aged *D. odorifera* individuals is primarily attributed to illegal logging driven by the high economic value of its heartwood. The observed age structure, characterized by abundant young individuals and a severe lack of mature adults, superficially resembles a growing population. Based on the evidence of illegal logging traces found in the field, we propose a scientific hypothesis of ‘false increase’: that this apparent growing‐type structure may not represent genuine natural population growth, but rather a distorted structure caused by long‐term selective logging of mature individuals and the consequent persistent absence of older age classes. It should be noted that this study lacks quantitative data on logging intensity, stump density, historical population baselines, or logging frequency analysis. Therefore, the ‘false increase’ hypothesis remains a inferential proposition rather than a confirmed mechanism, and requires future validation through long‐term fixed‐point monitoring and quantified disturbance data. We present it here as a possible population dynamic mechanism to provide a reference for future conservation research on this species. Human disturbance can cause population density decline and recession (Mcevoy et al. 2006; Li, Sun, et al. 2018; Gong et al. 2022).

The static life table shows that the standardized number of survivors for *D. odorifera* decreases from 1000 in class I to 15 in class V, consistent with the natural senescence pattern of woody plants (Tuo et al. 2021; Xiao et al. 2025). However, its mortality parameters show stage specificity: the number of deaths in class II is 478, the mortality rate increases from 0.16 in class I to 0.57 in class II, and peaks at 0.91 in class IV, forming a bimodal mortality peak pattern. This differs from the single mortality peak observed in *Amygdalus ledebouriana* (Guan et al. 2023) and *Acer miaotaiense* (Wang et al. 2024), indicating that *D. odorifera* faces dual bottlenecks of natural competition and human disturbance. Life expectancy decreases from 1.87 in class I to 0.50 in class V, a trend consistent with *Quercus mongolica* and *Pinus koraiensis* (Zhang et al. 2014; Zhang et al. 2022), but the survival potential of young individuals is lower than that of *Picea crassifolia* (Xiao et al. 2025). Furthermore, the disappearance rate surges to 2.40 in class IV, and combined with evidence of illegal logging, confirms human activity as the bottleneck causing the loss of old‐aged individuals. Therefore, the maintenance of the *D. odorifera* population is constrained dually by high natural mortality at the early stage (class II) and illegal logging at the later stage (classes IV–V).

Studies on *Betula platyphylla* (Ge et al. 2025), *Rhododendron xiaoxidongense* (Li et al. 2025), and *Horsfieldia hainanensis* (Jiang et al. 2017) indicate that limitations such as light availability in the early stages hinder the transition of young individuals to older age classes. *D. odorifera* similarly faces obstacles in sapling transition: the community canopy is heavily covered by lianas, resulting in high canopy density and insufficient understory light, which affects photosynthesis, reproduction, and seed setting. Intraspecific and interspecific competition exacerbates the mortality of young individuals (Qiu et al. 2020; Zhang et al. 2022; Xiao et al. 2025).

In the population quantitative dynamics analysis, the adjacent class transition indices (V1–V4) and the population dynamic index *V*_*pi*_ are all positive, indicating a growing type structure in the absence of disturbance, similar to Picea purpurea (Zhao et al. 2020; Zhang et al. 2022). However, the corrected *V*_*pi*_ *'* is much smaller than *V*_*pi*_, and the maximum risk probability *P*_*max*_ = 20.00, indicating that the population is sensitive to disturbance, with a disturbance resistance weaker than Picea purpurea (Zhao et al. 2020) but stronger than *Amygdalus ledebouriana* (Guan et al. 2023). The survival function decreases continuously, the cumulative mortality rate increases, the mortality density shows a small peak at class II, and the hazard rate approaches 1.0 at class IV, forming a pattern of high early mortality density and extremely high late‐stage hazard rate. This differs from *Populus wulianensis* (Wu et al. 2021), further confirming the dual‐stage limitations constraining the population.

Time series prediction shows that the number of individuals in all age classes is projected to increase in the future, with class V reaching 32 individuals after 5 age classes. This overall increasing trend is more optimistic than that predicted for *Pinus koraiensis* (Zhang et al. 2022), and is strongly supported by the sufficient reserve of individuals in class I, a mechanism consistent with *Picea crassifolia* (Xiao et al. 2025). However, it must be emphasized that this prediction was made using the moving average method to quantify the intrinsic recovery potential under an ideal conservation scenario (i.e., no illegal logging, stable habitat, and constant recruitment). In reality, the wild *D. odorifera* population is disturbance‐sensitive (*P*_max_ = 20 *P*_max_ = 20), and ongoing illegal logging coupled with the high juvenile mortality bottleneck would break these assumptions, making the optimistic growth trend unlikely to be achieved. Moreover, the extremely small sample size of mature individuals results in wide 95% confidence intervals for the projected values of older age classes, so the long‐term prediction can only indicate a directional trend of recovery rather than provide precise quantitative forecasts. Therefore, the core value of this prediction lies in revealing the species' natural recovery potential and providing a theoretical basis for implementing stage‐specific conservation strategies (i.e., completely curbing illegal logging and overcoming the juvenile survival bottleneck). This indicates that the *D. odorifera* population has good recovery potential if the bottlenecks at classes I‐II and IV‐V can be overcome.

Study limitations. Although this survey systematically sampled > 90% of the known distribution points of *D. odorifera* on Hainan Island using stratified typical sampling, which is suitable for analyzing the structure of a rare and endangered species, several limitations should be acknowledged. The extremely small number of mature individuals (only one in age class V) introduces statistical uncertainty in long‐term recovery predictions beyond five age‐class intervals. Moreover, the stratified typical sampling method, while effective for population structure analysis, has inherent limitations in accurately estimating the total population size across the entire island. In addition, the “space‐for‐time substitution” method for age estimation inevitably carries uncertainty. Nevertheless, these limitations do not affect the core conclusion regarding population regeneration potential based on the abundant young individual reserve.

## 5 Conclusion

In conclusion, the current growing‐type structure of *D. odorifera* may be interpreted under the ‘false increase’ hypothesis, i.e., it could result from the accumulation of surviving saplings following the illegal logging of mature adults, with human disturbance causing a population structural discontinuity. However, due to the lack of quantitative disturbance indicators (e.g., logging intensity, stump density, historical baseline), this hypothesis requires further testing with long‐term monitoring data. Nevertheless, the population is disturbance‐sensitive and possesses recovery potential, as confirmed by the demographic analyses. Population survival faces dual constraints: high mortality in low‐age classes and vulnerability of old‐aged individuals to illegal logging, making it a disturbance‐sensitive species. Dynamic prediction shows the population possesses recovery potential. Therefore, the conservation of *D. odorifera* should focus on overcoming these dual bottlenecks through staged interventions: (1) Improve understory light conditions by removing canopy lianas and dominant competing shrubs; (2) Establish real‐time monitoring systems and strengthen regular patrols to prevent illegal logging; (3) Implement rejuvenation measures for individuals weakened by liana infestation, such as pruning withered branches and leaves and applying artificial fertilization; (4) For small groups outside protected areas, establish conservation plots, conduct long‐term monitoring, and assess the impact of the false increase on the population's genetic potential.

## Author Contributions

**Chumin Ye:** data curation (lead), formal analysis (lead), investigation (lead), methodology (lead), software (lead), visualization (lead), writing – original draft (lead), writing – review and editing (lead). **Kai Zhang:** data curation (equal), formal analysis (equal), investigation (equal), methodology (equal), software (equal), writing – review and editing (equal). **Yukai Chen:** funding acquisition (lead), methodology (equal), writing – review and editing (equal). **Yongkang Dai:** investigation (equal), writing – review and editing (equal). **Liu Chen:** investigation (equal), writing – review and editing (equal).

## Funding

This work was supported Hainan Province Science and Technology Special Fund, ZDYF2023RDYL01, Hainan Institute of National Park (HINP), KY‐24ZK02, and Hainan Provincial Natural Science Foundation for Young Scholars: Research on the Origin, Impacts and Countermeasures of Forest Fragmentation of Hainan Tropical Rainforest National Park, 423QN234.

## Conflicts of Interest

All authors declare no potential conflicts of interest with respect to the research, authorship, and/or publication of this article. The funders (Hainan Province Science and Technology Special Fund, Hainan Institute of National Park) had no role in the design of the study; in the collection, analyses, or interpretation of data; in the writing of the manuscript; or in the decision to publish the results. No financial or personal relationships with other people or organizations that could inappropriately influence this work have been disclosed.

## Data Availability

The population individual and analysis data collected for the *Dalbergia odorifera* Population Study that support the findings of this study are openly available in the figshare repository at https://figshare.com/s/7f44e6d7db146cdc6965.
